# Towards engineering agaricomycete fungi for terpenoid production

**DOI:** 10.1093/jimb/kuaf020

**Published:** 2025-07-12

**Authors:** Riccardo Iacovelli, Dominik Mojzita, Peter Richard, Yvonne Nygård

**Affiliations:** VTT Technical Research Centre of Finland, 02150 Espoo, Finland; VTT Technical Research Centre of Finland, 02150 Espoo, Finland; VTT Technical Research Centre of Finland, 02150 Espoo, Finland; VTT Technical Research Centre of Finland, 02150 Espoo, Finland; Chalmers University of Technology, Department of Life Sciences, 412 96 Gothenburg, Sweden

**Keywords:** *Agaricomycetes*, Terpenoids, Biotechnology, Mushrooms, Cell factories

## Abstract

Since ancient times, humans have harnessed the vast metabolic abilities of fungi to produce food, beverages, and medicines. Biotechnology and genetic engineering have opened new avenues to tailor and enhance these abilities, transforming fungi into powerful industrial workhorses.

In this minireview, we focus on the biotechnological potential of *Agaricomycetes*, a class of basidiomycete fungi that includes the so-called 'true' mushrooms. Although many species are widely used in the food sector, their broader potential in biotechnology remains largely untapped. These fungi naturally produce a diverse array of metabolites with promising applications across various industries. Here, we highlight their ability to synthesize a wide range of terpenoids, many unique to this taxon, and we present recent advancements in genomics and genetic engineering tools developed for *Agaricomycetes*. We anticipate that continued progress in tailored genetic engineering tools and improved cultivation technologies will facilitate the establishment of these fungi as robust cell factories for producing valuable terpenoids, with significant contributions to the food, biotech, and pharmaceutical sectors.

**One-Sentence Summary**: This minireview highlights the potential of mushroom-forming fungi to be engineered into cell factories for producing terpenoids—valuable compounds with diverse applications in food, medicine, and biotechnology.

## Graphical Abstract

**Graphical Abstract:**
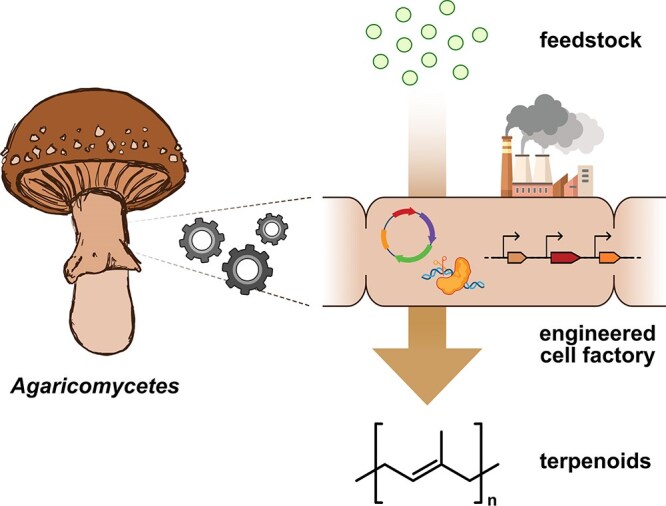

## Introduction

Fungi were among the first complex organisms to make land, and it is now a broadly accepted view that they helped plants establish themselves in terrestrial habitats and essentially kickstart life as we know it (Jermy, 2011). Throughout our planet's history, fungi have thrived in all Earth's ecosystems. Therefore, it is not surprising that our relationship with them is thousands of years old. Microscopic fungi like baker's yeast and moulds (typically belonging to the *Ascomycota* division) have been used for centuries to produce food and beverages (Money, 2016). Modern fungal biotechnology was born in the 20th century, when food chemist James Currie pioneered a process to produce citric acid with the common mould *Aspergillus niger* (Cairns et al., 2018). Since then, ascomycete fungi have exponentially gained traction and became established industrial cell factories used to produce enzymes, food additives, pharmaceuticals, and more (Nielsen & Nielsen, 2017; Punt et al., 2002; Yang et al., 2017).

Macroscopic fungi from the *Basidiomycota* division have been equally important to mankind. Notably, nearly all edible mushrooms (aside from morels and truffles) belong to the class *Agaricomycetes* within this division. Besides their value as food, agaricomycete fungi are particularly attractive from a biotechnological perspective due to other features such as their unmatched ability to degrade lignin. Furthermore, *Agaricomycetes* produce a plethora of hydrolytic enzymes with numerous applications in industry (Janusz et al., 2017) and they can also be used to produce packaging and even construction materials (Meyer et al., 2020). Lastly, a wide variety of bioactive metabolites have been isolated from their fruiting bodies, in particular terpene and terpenoid compounds that are unique to *Agaricomycetes* (Gressler et al., 2021; Schmidt-Dannert, 2015). However, heterologous production hosts are often required to access valuable metabolites and enzymes from *Agaricomycetes*. Indeed, many mushroom species form symbiotic relationships with specific host trees (Sharma, 2017) and cannot be readily cultivated in laboratory conditions, nor genetically modified. Unfortunately, heterologous production of *Agaricomycetes* metabolites in well-established ascomycete hosts has so far proven very challenging. This may be due to differences in gene structure and genetic regulatory elements across taxa, abundance of proteases that degrade basidiomycete enzymes, limited availability of essential precursors (e.g. due to competing metabolic pathways), and the absence of appropriate auxiliary proteins and cellular machinery required for correct processing of the products (e.g. membrane transport and secretion) (Casado López et al., 2016; Fischer et al., 2016). Thus, it would be beneficial to establish genetically amenable *Agaricomycetes* hosts and expand the currently limited set of suitable genetic tools. Universal transformation protocols and synthetic biology tools, such as CRISPR-based systems and toolkits containing easy-to-assemble genetic parts (e.g. promoters, selection markers, reporters, etc.), need to be developed. These advancements are expected to pave the way for a broader application of *Agaricomycetes* in biotechnology and related industries.

## *Agaricomycetes* are Terpenoid Super-Producers

Fungi are incredible producers of secondary metabolites (SMs)—organic molecules not involved in primary metabolic processes such as growth or reproduction, but that play important roles in shaping interactions with other organisms and with the environment. At the time of writing, 23,040 unique SMs from fungi have been described and recorded in the Natural Product Atlas (van Santen et al., 2021). Of these, about 94% originate from fungi belonging to either *Ascomycota* (17,519, 76%) or *Basidiomycota* (4,206, 18%). In fungi, SMs are typically biosynthesized by specialized enzymes that are encoded by physically clustered sets of genes, so-called biosynthetic gene clusters (BGCs). These can be predicted directly from genetic sequences, which allows researchers to easily gauge the biosynthetic potential of an organism. That is, the specific set of BGCs that the genome encodes, which is directly correlated with the capacity of an organism to produce secondary metabolites. In accordance with the numbers mentioned above, kingdom-wide analyses suggest that the majority of the biosynthetic potential of fungi is concentrated within the two sister divisions *Ascomycota* and *Basidiomycota* (Mosunova et al., 2020; Zhang et al., 2024). However, it is worth mentioning that natural product research and fungal genomics have focused mainly on these two divisions, leaving others (e.g. *Zoopagomycota* and *Mucoromycota*) substantially under-sampled. Actual differences in abundance of BGCs across the fungal kingdom might thus be more nuanced. Even so, ascomycetes undoubtedly possess the highest number of BGCs per genome, with their biosynthetic repertoire largely represented by polyketides and non-ribosomal peptides. Instead, the genomes of basidiomycetes carry less BGCs in total, but they encode a higher number and a wider diversity of terpene BGCs (Mosunova et al., 2020; Zhang et al., 2024). This is particularly true for the *Agaricomycetes*, which produce many unique terpenoids (Table 1) (Schmidt-Dannert, 2015; Steindorff et al., 2024). While the term ‘terpenoids’ more accurately refers to oxygen-containing compounds derived from terpenes (which consist only of carbon and hydrogen atoms) (Moss et al., 1995), we use it throughout this article to collectively refer to both groups for simplicity.

**Table 1.** Examples of Terpenoids Produced by *Agaricomycetes* (Compounds Unique to This Taxon in Bold) With Applications in the Food, Pharmaceutical, and Chemical Industries

Name	Chemical structure	Type	Producer organisms	Applications/bioactivities
4-thujanol	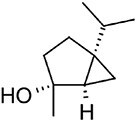	C10	*Amanita ovoidea* (Rapior et al., 1996)	Flavour and fragrance (Bhatia et al., 2008)
Cis-linalool oxide	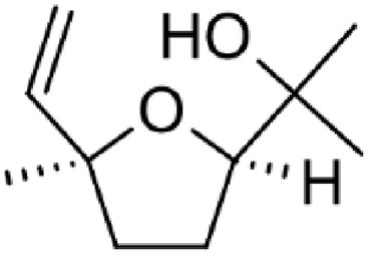	C10	*Clitocybe nebularis* (Rapior et al., 1996)	Flavour and fragrance (Maróstica & Pastore, 2007)
α-pinene	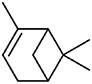	C10	*Amanita* spp. (Breheret et al., 1997)	Anti-inflammatory, antimicrobial, cytotoxic (Salehi et al., 2019)
**Hirsutenol F**	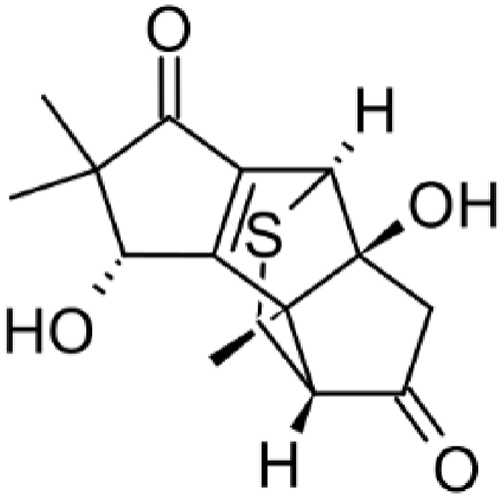	C15	*Stereum hirsutum* (Yoo et al., 2006)	Antioxidant (Yoo et al., 2006)
**Illudin M**	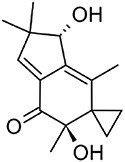	C15	*Omphalotus olearius* (Wawrzyn et al., 2012)	Cytotoxic (Schobert et al., 2011)
**Flammuspirone C**	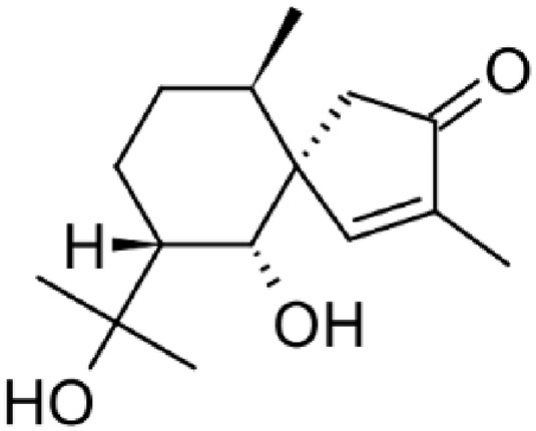	C15	*Flammulina velutipes* (Tao et al., 2016)	Antidiabetic, statin (Tao et al., 2016)
**Pleuromutilin**	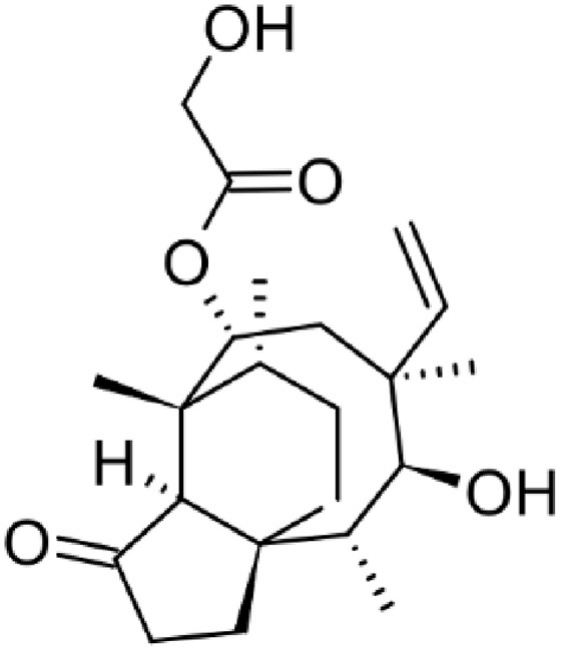	C20	*Clitopilus* spp. (Hartley et al., 2009)	Antibiotic (Hartley et al., 2009)
**Erinacine A**	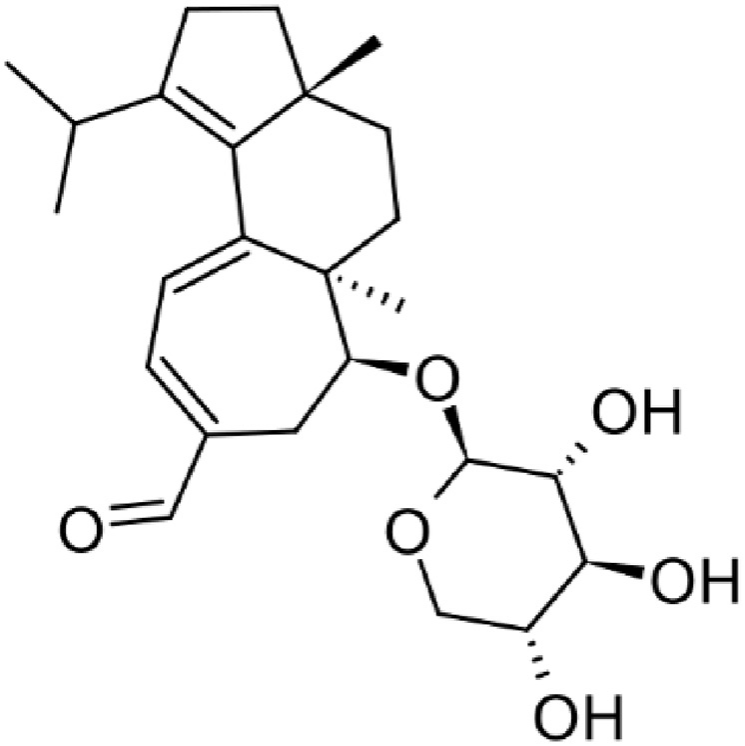	C20	*Hericium erinaceus* (Friedman, 2015)	Antioxidant, cytotoxic, neuro-protective (Friedman, 2015)
**Ganoderic acid A**	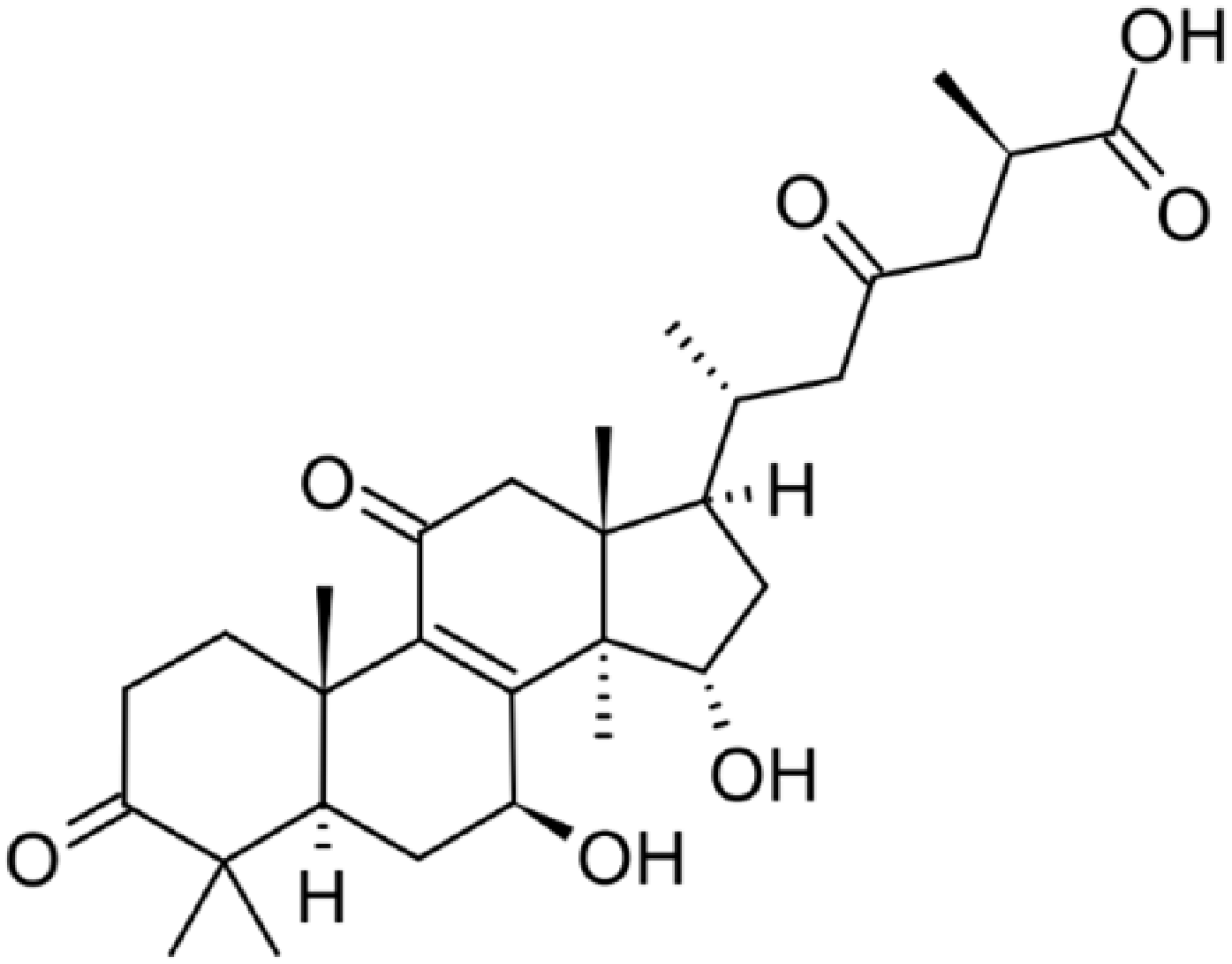	C30	*Ganoderma lucidum* (Paterson, 2006)	Anticancer (Jia et al., 2023)
**Inotodiol**	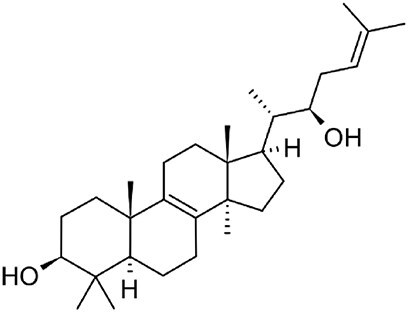	C30	*Inonotus obliquus* (L. Ma et al., 2013)	Anticancer, antiviral, anti-inflammatory, anti-allergic (Maza et al., 2021)
**Hericenone A**	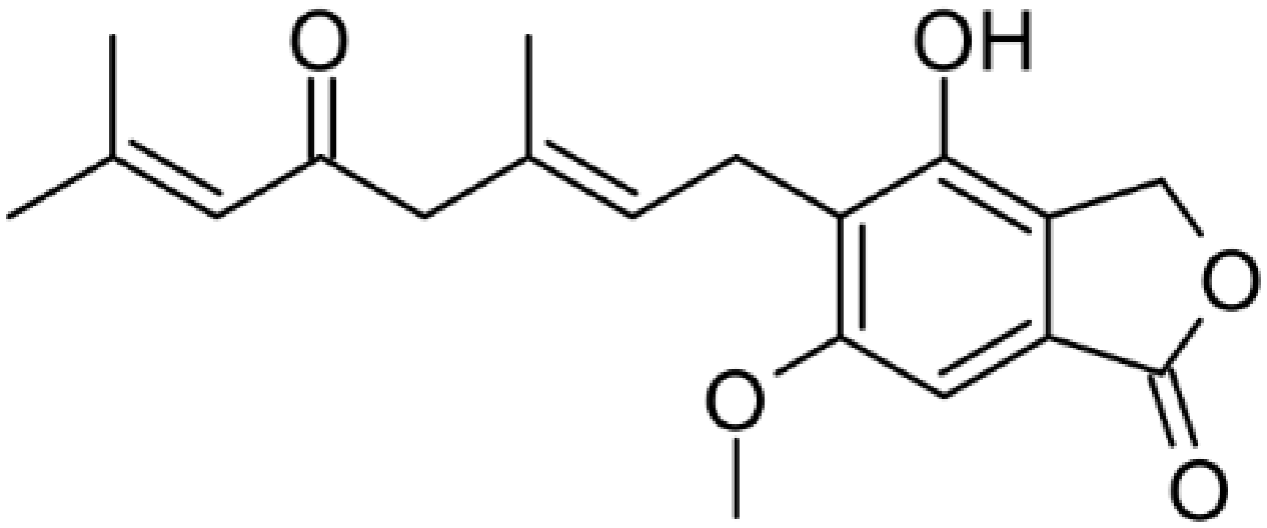	MT**^a^**	*Hericium erinaceus* (Friedman, 2015)	Cytotoxic (B.-J. Ma et al., 2010)
**Terreumol A**	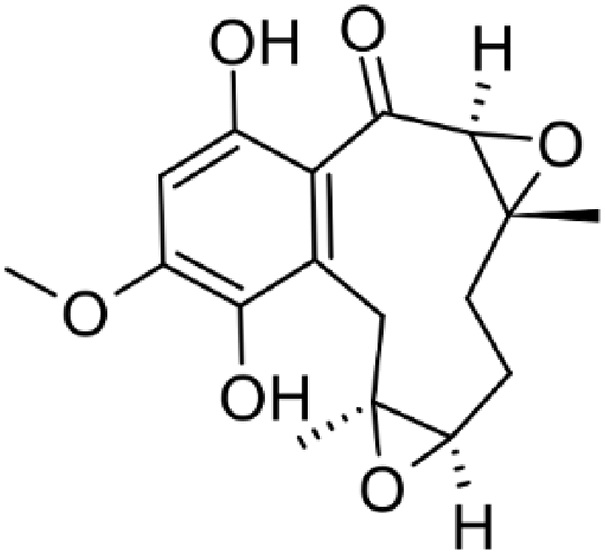	MT**^a^**	*Tricholoma terreum* (Yin et al., 2013)	Cytotoxic (Yin et al., 2013)
Cis-polyisoprene	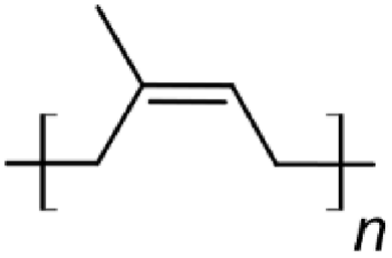	Cn**^a^**	*Lactarius volemus* (Ohya et al., 1998)	Natural rubber

a MT = meroterpenoids; Cn = polyisoprenes.

**Fig. 1.**
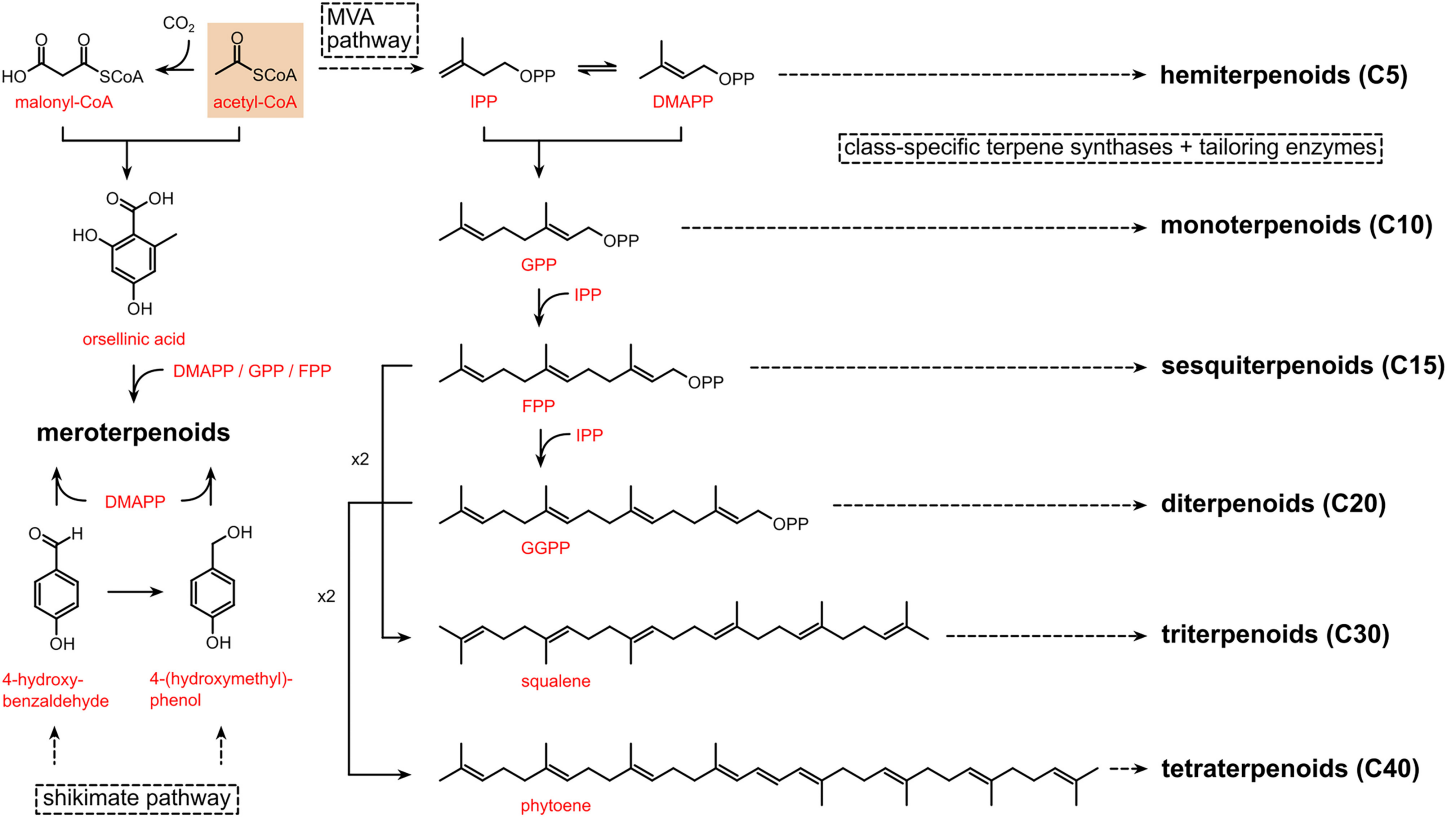
Major biosynthetic routes of terpenoids in *Agaricomycetes*. Acetyl-CoA is the central precursor for terpenoids in these fungi: it is used for the biosynthesis of the scaffold of polyketide-derived meroterpenoids, and to generate isoprene units IPP and DMAPP through the mevalonate (MVA) pathway. *Note*. IPP = isopentenyl pyrophosphate; DMAPP = dimethylallyl pyrophosphate; GPP = geranyl pyrophosphate; FPP = farnesyl pyrophosphate; GGPP = geranylgeranyl pyrophosphate.

With over 80,000 known terpenoids, this is the largest and most diverse group of natural products (Luo et al., 2024). The core structure of all terpenoids derives from the condensation of the functional 5-carbon isoprene units isopentenyl pyrophosphate (IPP) and dimethylallyl pyrophosphate (DMAPP). Based on the number of condensation reactions, and the resulting length of their linear precursors, terpenoids can be classified into several groups (Fig. 1). The biosynthesis of different types of terpenoids is achieved through the activity of class-specific synthases and/or cyclases such as sesquiterpene synthases, diterpene synthases, and oxidosqualene/lanosterol cyclases (Pan et al., 2024; Yang et al., 2017). These are further diversified by a variety of tailoring enzymes, such as P450 monooxygenases and reductases. Examples from all major terpenoid groups shown in Fig. 1 have been found in *Agaricomycetes*, with sesquiterpenoids (C15) being particularly abundant and diverse (Gressler et al., 2021; Schmidt-Dannert, 2015; Wang et al., 2021). Humulyl-derived bioactive sesquiterpenoids (such as hirsutane and illudanes) from *Agaricomycetes* are an especially attractive group because they show potent anti-inflammatory, cytotoxic, antiviral, and antibacterial activities (Schmidt-Dannert, 2015). In addition, several bioactive meroterpenoids have been isolated from *Agaricomycetes*, including medicinal fungi such as *Ganoderma* and *Hericium* (Friedman, 2015; Li et al., 2015; Niu et al., 2006; Wang et al., 2020). Meroterpenoids are diverse molecules with a partial terpenoid structure, and in agaricomycete fungi they are often functionalized phenolics and orsellinic acid derivatives, such as hericenones and erinacerins (Friedman, 2015), terreumol (Yin et al., 2013), and cloquetin (Braesel et al., 2017). Unique cytotoxic and anti-inflammatory lanostane-triterpenoids (C30) such as ganorbiformins (Isaka et al., 2013), ganoderic acids (Paterson, 2006), and inotodiol (Ma et al., 2013) have also attracted considerable attention. Lastly, ectomycorrhizal species of the genus *Lactarius* have long been known to produce large amounts of cis-polyisoprene, a natural rubber with similar properties to that extracted from the rubber tree (Ohya et al., 1998; Tanaka et al., 1994).

Interestingly, most building blocks necessary for the construction of terpenoid compounds in *Agaricomycetes* ultimately derive from the central metabolite acetyl-CoA. While secondary metabolism in ascomycetes channels acetyl-CoA almost entirely to polyketides biosynthesis routes, agaricomycete fungi instead redirect it to terpene synthesis (Mosunova et al., 2020; Schmidt-Dannert, 2015; S. Zhang et al., 2024). This suggests that—though both *Ascomycota* and *Basidiomycota* share central metabolic routes and possess abundant pools of acetyl-CoA—they evolved different chemical arsenals to mediate interactions which are specific to their ecological niches. *Agaricomycetes* are therefore specialized terpenoid producers, which makes them particularly attractive for the biotechnological production of bioactive terpenoids and terpenoid-based materials.

## Genetic Engineering of *Agaricomycetes*

**Fig. 2.**
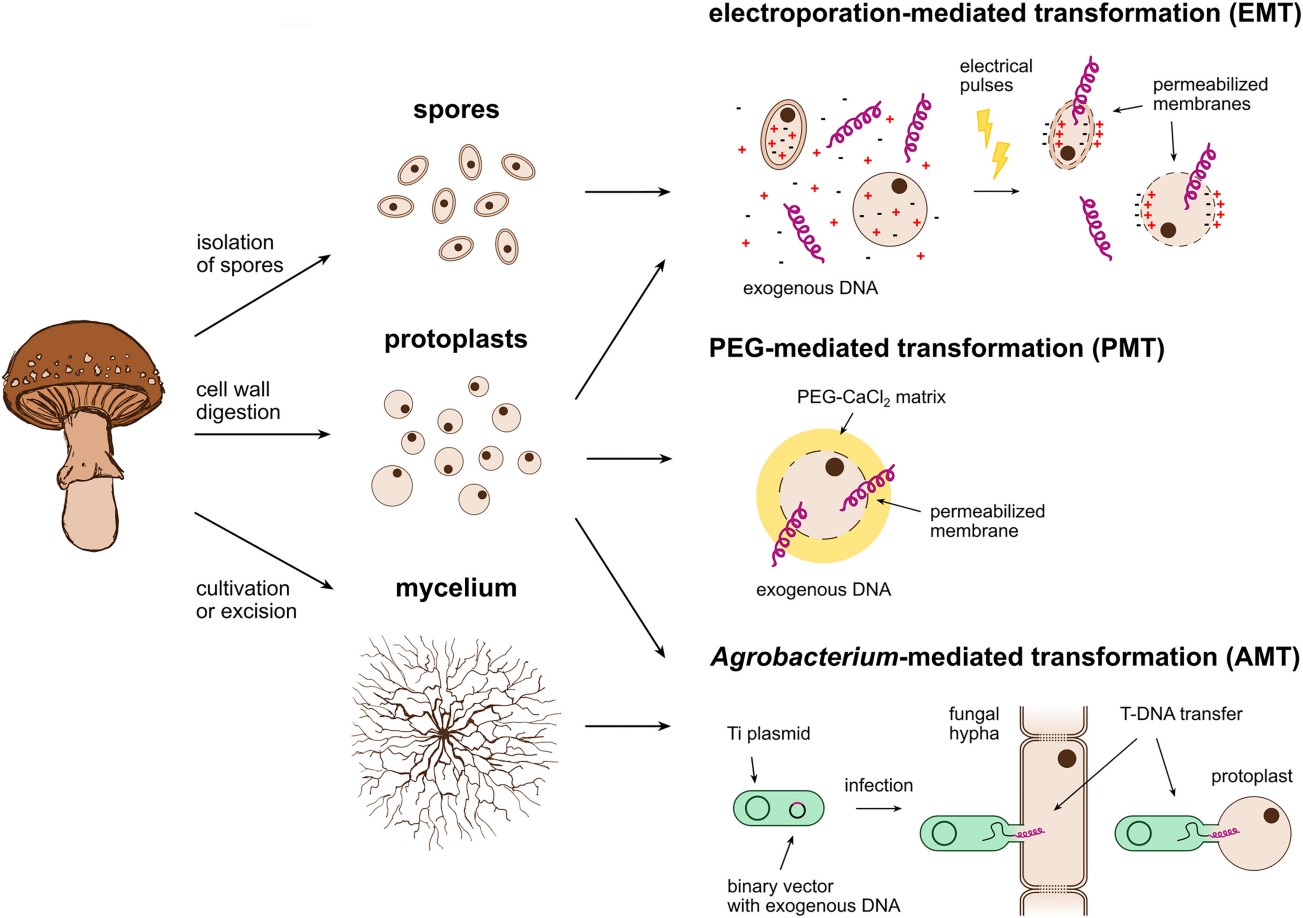
Established methods for genetic transformation of *Agaricomycetes*. Different types of fungal cells and tissues can be used as recipient material depending on the fungal species.

Several methods for genetic transformation of fungi have been established, the most common being PEG-mediated (PMT) (Case et al., 1979), electroporation-mediated (EMT) (Chakraborty & Kapoor, 1990), and *Agrobacterium*-mediated transformation (AMT) (Michielse et al., 2008). PMT requires the enzymatic digestion of the cell wall to produce fungal protoplasts. Polyethylene glycol and divalent cations (typically Ca^2+^) are then used to destabilize the plasma membrane of the protoplasts and facilitate DNA uptake. Electroporation employs rapid electric pulses to create temporary pores in the plasma membrane, although the exact mechanisms underlying DNA uptake during EMT are poorly understood. AMT makes use of the native ability of the soil bacterium *Agrobacterium tumefaciens* to transfer DNA into wounded plant cells. Domesticated strains of *Agrobacterium* are used for AMT in combination with modified plasmids that promote DNA transfer into fungal cells (Fig. 2). Physical methods such as biolistic DNA delivery (Lorito et al., 1993) and shock-wave–mediated transformation (Magaña-Ortíz et al., 2013) have also been reported for some ascomycetes.

The development of efficient genetic transformation systems in *Agaricomycetes* has proven more challenging compared to ascomycetes. Nonetheless, a few agaricomycete fungi have been genetically manipulated, including the well-studied *Ganoderma lucidum, Pleurotus ostreatus*, and *Schyzophyllum commune* (Salame et al., 2012; L. Sun et al., 2001; Van Peer et al., 2009). EMT, PMT, and AMT have all been used in *Agaricomycetes*, although these methods typically need to be extensively optimized and tailored to each specific species. This is likely due to several factors, such as key differences in the composition of the fungal cell wall compared to ascomycetes (Gow et al., 2017) and heterokaryosis (Auxier et al., 2022). Each transformation method has benefits and drawbacks: EMT is a fast method, but it requires extensive parameter optimization and larger numbers of recipient cells, due to substantial cell death caused by the high voltages applied (Hu et al., 2014). AMT can be used with protoplasts or mycelia obtained from different starting material, such as germinating spores (Ford et al., 2016), mycelial pellets (Hanif et al., 2002), and fruiting body fragments (Shi et al., 2017). AMT generally yields genetically stable transformants, but it is severely limited by complex and long workflows. Furthermore, its success can depend on many variables such as co-culturing conditions and the choice of an appropriate *Agrobacterium* strain (Li et al., 2017). PMT, on the other hand, is cheap, has good transformation efficiencies, and follows rather standard procedures. Therefore, it is often the first approach that is attempted for the transformation of new fungal species. Furthermore, PMT enables CRISPR-based genome engineering using pre-assembled ribonucleoparticles (RNPs) that can be delivered directly into the fungal protoplasts (Boontawon, Nakazawa, Xu, et al., 2021). PMT is nonetheless unfortunately limited to the species for which protoplasts can be generated (Table 2). Most of the successful examples of genetic transformation in *Agaricomycetes* reported the use of protoplasts as recipient cells. The main advantage of using protoplasts is their lack of cell wall, which enables more efficient uptake of exogenous DNA. Indeed, protoplasts have also been used in combination with other DNA delivery methods. For example, protoplasts of *Agaricus bisporus* (van de Rhee et al., 1996), *Ganoderma lucidum* (Sun et al., 2001), and *Flammulina velutipes* (Kim et al., 2010) have been successfully engineered via electroporation. Lastly, AMT of protoplasts has been reported in *F. velutipes* (Shi et al., 2017) and *Hypsizygus marmoreus* (Zhang et al., 2014). However, determining the right conditions and developing protocols to generate protoplasts of new species can be tedious, and commercially available cell-wall degrading enzymes often prove ineffective. Therefore, expanding our knowledge of cell wall biology in *Agaricomycetes* is essential to identify key factors for protoplast formation. This will lead to the development of universal tools and procedures, enabling efficient genetic transformation across multiple agaricomycete species (Li et al., 2017).

**Table 2.** Protoplast Preparation Conditions for Agaricomycete Fungi

Species	Starting material	Lytic enzyme(s)^a^	Digestion buffer	Protoplasts yield	Ref.
*Pleurotus ostreatus*	Mycelium	2% lysing enzymes from *T. harzianum*, 0.05% chitinase from *T. viride*	0.5 M sucrose	5 × 10^7^/mL**^b^**	Salame et al. (2012)
*Ganoderma lucidum*	Mycelium	1.5% lywallzyme	0.6 mannitol, 100 mM Na citrate	5 × 10^7–^5 × 10^8^/mL**^b^**	Sun et al. (2001)
*Schizophyllum commune*	Mycelium	0.15% lysing enzymes**^c^**	1 M MgSO_4_,10 mM malate	1 × 10^8^/mL**^b^**	Van Peer et al. (2009)
*Coprinopsis cinerea*	Mycelium or oidia	2% Cellulase Onozuka R-10, 0.1% chitinase	0.5 M mannitol, 50 mM maleate	1–5 × 10^8^/mL	Binninger et al. (1987)
*Coprinellus congregatus*	Mycelium	0.2% Novozyme 234	0.5 M MgSO_4_, 50 mM maleate	1.25 × 10^8^/mL**^b^**	Leem et al. (1999)
*Ceriporiopsis subvermispora*	Mycelium	0.25% lysing enzymes from *T. harzanium*, 0.5% zymolase, 0.1% chitinase	0.5 M mannitol, 25 mM maleate	2 × 10^7^/mL^b^	Honda et al. (2019)
*Clitopilus passeckerianus*	Mycelium	5% lysing enzymes from *T. harzanium*	1 M MgSO_4_, 0.6 M phosphate	1 × 10^8^/mL^b^	Kilaru et al. (2009)
*Trametes versicolor*	Mycelium	0.2% Novozyme 234	0.5 M MgSO_4_, 50 mM maleate	n/a	Kim et al. (2002)
*Lentinula edodes*	Mycelium	2.5% cellulase Onozuka RS, 0.1 % chitinase	0.6 mannitol, 50 mM succinate	1 × 10^8^ per 1 g wet biomass	Irie et al. (2003)
*Cyclocybe aegerita*	Oidia	0.1% lysing enzymes from *T. harzianum*, 0.1% yatalase	1.2 M MgSO_4_, 50 mM maleate	0.9–1.2 × 10^8^/mL**^b^**	Herzog et al. (2019)
*Ganoderma multipileum*	Mycelium	0.75% lysing enzymes from *T. harzianum*	0.6 M sucrose in K Phosphate**^d^**	3 × 10^7^/mL	Chou & Tzean (2016)
*Pleurotus eryngii*	Mycelium	3% yatalase, 4.5% lysing enzymes from *T. harzianum*	1.2 M MgSO_4_, 10 mM Na Phosphate	n/a	Wang et al. (2021)
*Dichomitus squalens*	Mycelium	2% yatalase	0.6 M MgSO_4_, 50 mM maleate	2 × 10^6^ per 1 g wet biomass	Daly et al. (2017)

a Concentration of lytic enzymes in digestion buffer is expressed in weight/volume % (10 mg/mL = 1%).

b Protoplasts yield not directly reported. Numbers shown refer to the concentration of protoplasts used for each PEG-mediated transformation.

c Organism of origin of lytic enzymes not specified by source institution.

d Concentration of buffering agent potassium phosphate not specified.

## Latest Advancements in Genomics and CRISPR-Based Genome Editing of *Agaricomycetes*

Genetic engineering of fungi (and of organisms in general) to study gene function or to enable biotech applications requires technologies that allow precise manipulation of DNA sequences. These include, for example, targeted deletions of genes of interest (Santiago et al., 2008), functional expression of heterologous genes, or selected modifications of native metabolic pathways (Cho et al., 2022). These technologies depend, first and foremost, upon the availability of high-quality genome sequencing data. Fortunately, dramatic reductions in costs and technological advancements in the last decade—particularly the development and spread of long-read sequencing platforms—have made it relatively easy to obtain complete high-quality assemblies of many fungal genomes (Agustinho et al., 2024). The number of genomes in the JGI MycoCosm database alone has multiplied tenfold in the past 10 years, reaching 2,650 at the start of 2025 (Grigoriev et al., 2014). Combined with those in the NCBI genome database (www.ncbi.nlm.nih.gov/genome), a total of 23,105 fungal genomes are available at the time of writing (Table 3).

**Table 3.** Number of Fungal Genomes Available on Online Databases in 2025

Databases	Fungi	*Ascomycota*	*Basidiomycota*	(*Agaricomycetes*)	Others
MycoCosm	2,650	1,690	679	528	281
NCBI Genome	20,455	16,530	2,971	1,644	954
Total**^a^** (%)	23,105	18,220 (78.9 %)	3,650 (15.8 %)	2,172 (9.4 %)	1,235 (5.35 %)

a Eight hundred fifty-nine entries present in the NCBI database were submitted by JGI and potentially overlap.

Notably, despite ascomycetes continuing to receive the most attention, 2,172 of these genomes belong to *Agaricomycetes*, and the number keeps growing. This facilitates genetic research in these organisms and plays a critical role in bioinformatics studies aimed at deciphering biosynthetic pathways of interest. For example, high-quality genome sequences and annotations were instrumental in elucidating the complete pathways of erinacines (Liu et al., 2019) and pleuromutilin (Wen et al., 2025), identifying key enzymes in the biosynthesis of ganoderic acids (Yuan et al., 2022), and characterizing the first steps of elusive meroterpenoid-producing pathways in *Stereum* and *Hericium* species (Braesel et al., 2017; Iacovelli et al., 2024).

The availability of high-quality genomic data is also essential for the establishment of advanced genome engineering tools such as CRISPR-based systems, as accurate sequence information is required to design guide RNAs and for targeted gene modifications. The first application of CRISPR-Cas9 in an agaricomycete fungus was described in 2017 when a tailored plasmid-based system was used to knock-out the *GFP* gene in a stable *GFP* expression line of *Coprinopsis cinerea* (Sugano et al., 2017). In plasmid-based systems, the codon-optimized Cas9 and guide RNA(s) genes are typically placed under the control of strong promoters on the same vector and co-expressed in vivo (Boontawon, Nakazawa, Inoue, et al., 2021; Nakazawa et al., 2022). Alternatively, the Cas9 system can be delivered via pre-assembled RNPs directly into the fungal protoplasts (Boontawon, Nakazawa, Choi et al., 2023). In both cases, Cas9 needs to be fused to an appropriate nuclear localization signal so that it can effectively localize into the nuclei to access the genomic target (Pohl et al., 2016). Typically, the use of pre-assembled RNPs leads to faster results thanks to their immediate cleavage activity upon transformation. A further advantage of RNPs-based approaches is that they do not require selection pressure to be maintained to ensure that Cas9 remains active at sufficient levels, as plasmid-based systems do. Additionally, continuous plasmid-based expression of Cas9 can increase toxicity and off-target activity. These unwanted side effects are considerably lower with the use of Cas9 RNPs, which are quickly degraded during natural protein turnover (Wang & Coleman, 2019).

CRISPR-Cas systems have been developed for several *Agaricomycetes*, including multiple species of *Ganoderma* (Qin et al., 2017; Tu et al., 2021) and *Pleurotus* (Boontawon et al., 2023; Wang et al., 2021), *Lentinula edodes* (Kamiya et al., 2023), *Agaricus bisporus* (Choi et al., 2023), and *Schyzophyllum commune* (Jan Vonk et al., 2019). CRISPR-based techniques offer a promising solution to improve the efficiency of targeted gene deletions and insertions, which are typically inefficient in agaricomycete fungi due to their strong preference towards non-homologous end joining (NHEJ) over homologous recombination (HR) as DNA repair mechanism (Kim et al., 2015). For example, CRISPR-Cas has successfully been used for the targeted disruption of the orotidine-5′-monphosphate decarboxylase-encoding *pyrG* gene in several species (Boontawon, Nakazawa, Inoue, et al., 2021; Eom et al., 2023; Liu et al., 2022). The resulting strains are auxotrophic for uracil and uridine, while also exhibiting resistance to 5-fluoroorotic acid. This dual positive/negative selection system has been widely applied in the genetic engineering of ascomycete fungi, and it is particularly useful as it enables marker recycling and sequential transformations through the use of ‘self-excising’ cassettes (Maruyama & Kitamoto, 2008). Establishing this system in *Agaricomycetes* will undoubtedly facilitate genetic engineering efforts in these fungi. CRISPR systems can also be used to generate NHEJ-deficient strains by deleting genes that encode for NHEJ-associated DNA repair proteins, such as the *ku70* homolog (Tu et al., 2021), thereby increasing the probability of HR-mediated events and success rate of targeted modifications. Combining such host strains with CRISPR-based tools in subsequent transformation rounds can yield remarkably high editing efficiencies (Jan Vonk et al., 2019; Tu et al., 2021). For example, a Δ*ku70* mutant of *Ganoderma lucidum* showed 96.3 and 93.1% frequencies of targeted gene insertion and replacement, respectively, using a *ura3* (*pyrG* homolog) expression cassette as donor DNA (Tu et al., 2021).

## *Agaricomycetes* as Cell Factories

So far, most examples of genetic engineering in *Agaricomycetes* concern proof-of-concept studies, for example insertion of a selection marker cassette alone or expression of reporter genes such as GFP (Burns et al., 2005; Ford et al., 2016; Sun et al., 2001). Such studies are particularly useful to provide crucial insights into the mechanisms of gene expression. Notably, it has been shown that the presence of introns may be necessary for the successful expression of genes of interest in several *Agaricomycetes* (Burns et al., 2005; Ford et al., 2016; Lugones et al., 1999). Currently, only a limited number of studies have demonstrated successful engineering of *Agaricomycetes* to overproduce enzymes or other valuable metabolites (Table 4). These efforts have primarily focused on hydrolytic enzymes such as laccases and cellulases, which have applications across several industries (e.g. textile, food, and paper). More recently, the first examples of engineering *Agaricomycetes* for terpenoid production have been reported. For instance, homologous overexpression of core mevalonate and terpenoid pathway genes has been used to generate *Ganoderma lucidum* strains that produce increased titers of ganoderic acids (Fei et al., 2019; Zhang et al., 2017). Similarly, homologous overexpression of the key pathway genes geranylgeranyl diphosphate synthase and pleuromutilin-specific cyclase in the native producer *Clitopilus passeckerianus* led to increased pleuromutilin titers of 6.9 g/L (Wen et al., 2025).

**Table 4.** Examples of Genetically Engineered *Agaricomycetes* Cell Factories

Organism(s)	Strategy	Product(s)	Reference
*Trametes versicolor*	Overexpression of native laccase *cvl3* gene	Laccase	Kajita et al. (2004)
*Pycnoporus cinnabarinus*	Overexpression of native laccase *lac1* gene	Laccase	Alves et al. (2004)
*Volvariella volvacea*	Heterologous expression of multi-functional cellulase *mfc* gene from *Pomacea maculata*	Fruiting bodies yield	Zhao et al. (2010)
*Coprinopsis cinerea*	Overexpression of native laccase *Lcc5* gene + silencing (RNAi) of native chitinase *ChiE2* gene	Laccase	Yao et al. (2024)
*Ganoderma lucidum*	Overexpression of native nicotinamide mononucleotide adenyltransferase *nmnat* gene	Cellulase	Wang et al. (2020)
*Ganoderma lucidum*	Overexpression of native farnesyl pyrophosphate synthase *FPS* gene	Ganoderic acids	Fei et al. (2019)
*Ganoderma lucidum*	Overexpression of native HMG-CoA reductase *HMGR* and squalene epoxidase *SQLE* genes	Ganoderic acids	Zhang et al. (2017)
*Clitopilus passeckerianus*	Overexpresion of native geranylgeranyl diphosphate synthase *ple-ggpps* and diterpene cyclase *ple-cyc* genes	Pleuromutilin	Wen et al. (2025)

One of the main limitations to the application of higher fungi as cell factories is their complex morphology under submerged cultivation conditions typically used in industrial processes (Meyer, 2021). Fungi are major decomposers and as such they typically favour growth on substrate. When cultivated in liquid media, they often form big clumps or pellets that may have negative effects on biomass accumulation. On the other hand, some species can grow very rapidly as dispersed hyphae, which can cause the cultivation medium to become highly viscous and limit gas-liquid exchanges, and mechanically hamper fermentor parts (Cairns et al., 2019). A limited number of studies show that different morphology types might be desirable for different products. Small pellets might lead to higher production and secretion of secondary metabolites (Cairns et al., 2019), whereas dispersed growth and larger amounts of biomass might work better for the extraction of cell wall components such as glucans (Berovic, 2023). Unfortunately, the progress with characterizing and influencing cultivation morphology has been limited to industrial ascomycete hosts such as *Penicillium, Aspergillus*, and *Trichoderma* species. For these fungi, it has been shown that varying inoculum size, medium composition and pH, or physical parameters such as agitation and aeration rates, are all viable routes to achieve a desired morphology (Cairns et al., 2019; Meyer, 2021). So far, fermentative processes with *Agaricomycetes* have been limited to well-studied species like *Ganoderma lucidum* and *Pleurotus ostreatus*, which can grow relatively well in submerged cultivations and reach biomass yields between 20 and 40 g/L in stirred tank reactors (Berovic, 2023). These species should serve as models to enhance our understanding of how cultivation conditions impact the morphological development and productivity of *Agaricomycetes* cell factories. Genetic engineering strategies will help uncover the underlying molecular mechanisms and apply this knowledge to fine-tune morphology for specific biotechnological processes.

## The Potential of *Agaricomycetes* for the Biotechnological Production of Terpenoids

The global market for terpenoids is predicted to surpass USD 1.6 billion by 2032, with a compound annual growth rate of 8.26% in the 2024–2032 period (valuemarketresearch.com/report/terpenes-market/, released in January 2025), highlighting the economic potential of these compounds. Terpenoids are used in several products, such as food and beverages, cosmetics, pharmaceuticals, and materials (Leavell et al., 2016). According to the report mentioned above, the most important terpenoids on the market are natural rubber and monoterpenoids used in the food and fragrance industries, such as limonene and linalool. These compounds are currently extracted from their natural plant sources, which limits their supply. While fermentative production can bypass this limitation, terpenoids biosynthesis pathways cannot always be reconstituted in current microbial hosts. Only a few terpenoids have been produced commercially by engineered microbes, most notably β-farnesene and artemisinic acid in engineered yeast strains (Leavell et al., 2016). In many other cases, the production yields are very low and not compatible with commercial processes (Xiao & Zhong, 2016). Additionally, many bioactive terpenoids with potential applications as pharmaceuticals can be toxic to current hosts, which limits compound discovery and greatly reduces the range of compounds that can be produced.

*Agaricomycetes* are naturally wired to produce commercial terpenoids in larger amounts, for example monoterpenoid aromas (Breheret et al., 1997) and natural rubber, which can reach up to 7% of the dry weight in certain species of *Lactarius* (Tanaka et al., 1994). Although commercial-scale production of terpenoids using agaricomycete fungi has not yet been realized, small-scale studies demonstrate that certain species can produce native terpenoids in submerged cultures at yields ranging from 0.1 to 10 g/L. For example, bioreactor cultivations of *Hericium erinaceus* reached production of erinacine A of 192 ± 42 mg/L (Krzyczkowski et al., 2010). Similarly, *Ganoderma lucidum* produced up to 367 ± 17 mg/L of ganoderic acids in fed-batch fermentation (Tang & Zhong, 2002). Remarkably, an optimized fermentation process for *Clitopilus mutilus* resulted in pleuromutilin titers of 12 g/L (S. Sun et al., 2017). These examples illustrate the potential of agaricomycete fungi to achieve commercially relevant titers, while also highlighting the substantial room for improvement, which can be achieved through genetic engineering (Wen et al., 2025). Furthermore, *Agaricomycetes* produce and secrete a wide array of bioactive terpenoids, such as anticancer sesquiterpenoids of the illudane family (Schmidt-Dannert, 2015), neuroprotective, anticancer, and antioxidant diterpenes of the erinacine family (Friedman, 2015), and cytotoxic triterpenoids such as ganoderic acids (Paterson, 2006). These capabilities suggest that agaricomycete fungi possess inherent self-resistance mechanisms (Yılmaz et al., 2023) that make them ideal hosts for the discovery and production of otherwise cytotoxic compounds. Thus, it is plausible that engineered *Agaricomycetes* cell factories could serve as robust production platforms for commercially relevant terpenoids from diverse sources, including compounds unique to agaricomycetes that cannot be produced in other hosts. This would significantly advance the biotechnological production of terpenoids.

**Fig. 3.**
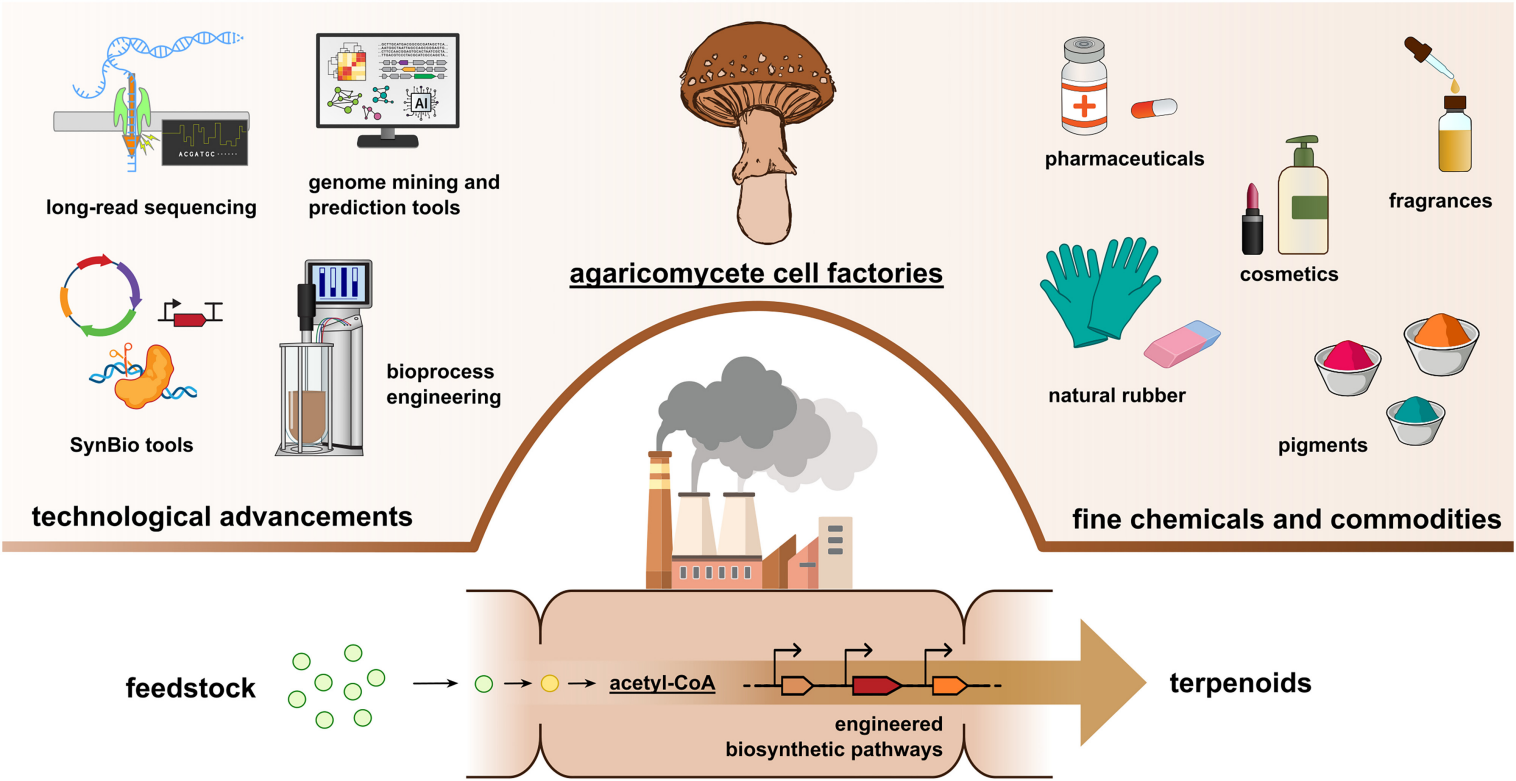
Envisioned development of *Agaricomycetes*-based cell factories for terpenoid production.

## Future Directions

With the continuous advancements in genetic engineering, synthetic biology, and cultivation technologies, it may be possible to establish *Agaricomycetes* alongside existing industrial hosts as effective cell factories for the biotechnological production of various goods. *Agaricomycetes* with enhanced biodegrading abilities could be used to efficiently recycle agricultural side streams and produce medicines or food (Ahlborn et al., 2019). Their fruiting bodies are already an important part of our diet (Rizzo et al., 2021). Therefore, one could envision engineering easy-to-cultivate ‘superfood mushrooms’ with higher contents of essential amino acids, vitamins, and fatty acids. These mushrooms could also serve as high-quality and sustainable animal feed, carving out a more significant role in supporting global food security. Importantly, engineered *Agaricomycetes* cell factories will provide a unique platform to produce supply limited terpenoids and discover new fungal terpenoids with applications in the food, cosmetics, pharmaceutical, and biotech industries (Fig. 3). Ultimately, *Agaricomycetes* provide a promising avenue for sustainable biotechnological innovations, strengthening the role of fungi in promoting a fully circular economy.

## Data Availability

All data underlying this article are available within the article.

## References

[bib1] Agustinho D. P., Fu Y., Menon V. K., Metcalf G. A., Treangen T. J., Sedlazeck F. J. (2024). Unveiling microbial diversity: Harnessing long-read sequencing technology. Nature Methods, 21(6), 954–966. 10.1038/s41592-024-02262-1 38689099 PMC11955098

[bib2] Ahlborn J., Stephan A., Meckel T., Maheshwari G., Rühl M., Zorn H. (2019). Upcycling of food industry side streams by basidiomycetes for production of a vegan protein source. International Journal of Recycling of Organic Waste in Agriculture, 8(S1), 447–455. 10.1007/s40093-019-00317-4

[bib3] Alves A. M. C. R., Record E., Lomascolo A., Scholtmeijer K., Asther M., Wessels J. G. H., Wösten M. A. B. (2004). Highly efficient production of laccase by the basidiomycete *Pycnoporus cinnabarinus*. Applied and Environmental Microbiology, 70(11), 6379–6384. 10.1128/AEM.70.11.6379-6384.2004 15528495 PMC525127

[bib4] Auxier B., Czaran T., Aanen D. K. (2022). Modelling the consequences of the dikaryotic life cycle of mushroom-forming fungi on genomic conflict. Elife, 11, 1–19. 10.7554/eLife.75917 PMC9084891 35441591

[bib5] Berovic M. (2023). Biochemical Engineering and Biotechnology of Medicinal Mushrooms(Berovic M., Zhong J.-J. (Eds.); Vol. 184). Springer International Publishing. 10.1007/978-3-031-36950-6

[bib6] Bhatia S. P., Letizia C. S., Api A. M. (2008). Fragrance material review on 4-thujanol. Food and Chemical Toxicology, 46(11), S295–S296. 10.1016/j.fct.2008.06.024 18640194

[bib7] Binninger D. M., Skrzynia C., Pukkila P. J., Casselton L. A. (1987). DNA-mediated transformation of the basidiomycete *Coprinus cinereus*. The EMBO Journal, 6(4), 835–840. 10.1002/j.1460-2075.1987.tb04828.x 3595558 PMC553472

[bib8] Boontawon T., Nakazawa T., Choi Y. J., Ro H. S., Oh M., Kawauchi M., Sakamoto M., Honda Y. (2023). Double-gene targeting with preassembled Cas9 ribonucleoprotein for safe genome editing in the edible mushroom *Pleurotus ostreatus*. FEMS Microbiology Letters, 370(February), 1–5. 10.1093/femsle/fnad015 36812945

[bib9] Boontawon T., Nakazawa T., Inoue C., Osakabe K., Kawauchi M., Sakamoto M., Honda Y. (2021). Efficient genome editing with CRISPR/Cas9 in *Pleurotus ostreatus*. AMB Express, 11(1), 30 10.1186/s13568-021-01193-w 33609205 PMC7897337

[bib10] Boontawon T., Nakazawa T., Xu H., Kawauchi M., Sakamoto M., Honda Y. (2021). Gene targeting using pre-assembled Cas9 ribonucleoprotein and split-marker recombination in *Pleurotus ostreatus*. FEMS Microbiology Letters, 368(13), 1–7. 10.1093/femsle/fnab080 34156066

[bib11] Braesel J., Fricke J., Schwenk D., Hoffmeister D. (2017). Biochemical and genetic basis of orsellinic acid biosynthesis and prenylation in a stereaceous basidiomycete. Fungal Genetics and Biology, 98, 12–19. 10.1016/j.fgb.2016.11.007 27903443

[bib12] Breheret S., Talou T., Rapior S., Bessière J.-M. (1997). Monoterpenes in the aromas of fresh wild mushrooms (Basidiomycetes). Journal of Agricultural and Food Chemistry, 45(3), 831–836. 10.1021/jf960417h

[bib13] Burns C., Gregory K. E., Kirby M., Cheung M. K., Riquelme M., Elliott T. J., Challen M. P., Bailey A., Foster G. D. (2005). Efficient GFP expression in the mushrooms *Agaricus bisporus* and *Coprinus cinereus* requires introns. Fungal Genetics and Biology, 42(3), 191–199. 10.1016/j.fgb.2004.11.005 15707840

[bib14] Cairns T. C., Nai C., Meyer V. (2018). How a fungus shapes biotechnology: 100 years of *Aspergillus niger* research. Fungal Biology and Biotechnology, 5(1), 13. 10.1186/s40694-018-0054-5 29850025 PMC5966904

[bib15] Cairns T. C., Zheng X., Zheng P., Sun J., Meyer V. (2019). Moulding the mould: Understanding and reprogramming filamentous fungal growth and morphogenesis for next generation cell factories. Biotechnology for Biofuels, 12(1), 1–18. 10.1186/s13068-019-1400-4 30988699 PMC6446404

[bib16] Casado López S., Sietiö O.-M., Hildén K., de Vries R. P., Mäkelä M. R. (2016). Homologous and Heterologous Expression of Basidiomycete Genes Related to Plant Biomass Degradation(pp. 119–160.). 10.1007/978-3-319-27951-0_5

[bib17] Case M. E., Schweizer M., Kushner S. R., Giles N. H. (1979). Efficient transformation of *Neurospora crassa* by utilizing hybrid plasmid DNA. Proceedings of the National Academy of Sciences, 76(10), 5259–5263. 10.1073/pnas.76.10.5259 PMC413120 159454

[bib18] Chakraborty B. N., Kapoor M. (1990). Transformation of filamentous fungi by electroporation. Nucleic Acids Research, 18(22), 6737. 10.1093/nar/18.22.6737 2147479 PMC332678

[bib19] Cho J. S., Kim G. B., Eun H., Moon C. W., Lee S. Y. (2022). Designing microbial cell factories for the production of chemicals. JACS Au, 2(8), 1781–1799. 10.1021/jacsau.2c00344 36032533 PMC9400054

[bib20] Choi Y. J., Eom H., Yang S. H., Nandre R., Kim S., Kim M., Oh Y. L., Nakazawa T., Honda Y., Ro H. S. (2023). Heterokaryosis, the main obstacle in the generation of PPO1-edited *Agaricus bisporus* by CRISPR/Cas9 system. Scientia Horticulturae, 318(April), 112095. 10.1016/j.scienta.2023.112095

[bib21] Chou T. H., Tzean S. S. (2016). Protoplasting, regeneration and transformation of medicinal mushroom *Ganoderma multipileum* using succinate dehydrogenase mutation gene as a selection marker. Annals of Microbiology, 66(1), 111–120. 10.1007/s13213-015-1087-0

[bib22] Daly P., Slaghek G. G., Casado López S., Wiebenga A., Hilden K. S., de Vries R. P., Mäkelä M. R. (2017). Genetic transformation of the white-rot fungus *Dichomitus squalens* using a new commercial protoplasting cocktail. Journal of Microbiological Methods, 143, 38–43. 10.1016/j.mimet.2017.10.001 28987554

[bib23] Eom H., Choi Y. J., Nandre R., Han H. G., Kim S., Kim M., Oh Y. L., Nakazawa T., Honda Y., Ro H. S. (2023). The Cas9-gRNA ribonucleoprotein complex-mediated editing of pyrG in *Ganoderma lucidum* and unexpected insertion of contaminated DNA fragments. Scientific Reports, 13(1), 1–8. 10.1038/s41598-023-38331-2 37429890 PMC10333205

[bib24] Fei Y., Li N., Zhang D. H., Xu J. W. (2019). Increased production of ganoderic acids by overexpression of homologous farnesyl diphosphate synthase and kinetic modeling of ganoderic acid production in *Ganoderma lucidum*. Microbial Cell Factories, 18(1), 1–9. 10.1186/s12934-019-1164-3 31253150 PMC6599323

[bib25] Fischer J., Schroeckh V., Brakhage A. A. (2016). Gene Expression Systems in Fungi: Advancements and Applications(Schmoll M., Dattenböck C. (Eds.)). Springer International Publishing. 10.1007/978-3-319-27951-0

[bib26] Ford K. L., Baumgartner K., Henricot B., Bailey A. M., Foster G. D. (2016). A native promoter and inclusion of an intron is necessary for efficient expression of GFP or mRFP in *Armillaria mellea*. Scientific Reports, 6(1), 1–10. 10.1038/srep29226 27384974 PMC4935854

[bib27] Friedman M. (2015). Chemistry, nutrition, and health-promoting properties of *Hericium erinaceus* (Lion's Mane) mushroom fruiting bodies and mycelia and their bioactive compounds. Journal of Agricultural and Food Chemistry, 63(32), 7108–7123. 10.1021/acs.jafc.5b02914 26244378

[bib28] Gow N. A. R., Latge J. P., Munro C. A. (2017). The fungal cell wall: Structure, biosynthesis, and function. The Fungal Kingdom, 5, 267–292. 10.1128/9781555819583.ch12 PMC11687499 28513415

[bib29] Gressler M., Löhr N. A., Schäfer T., Lawrinowitz S., Seibold P. S., Hoffmeister D. (2021). Mind the mushroom: Natural product biosynthetic genes and enzymes of basidiomycota. Natural Product Reports, 38(4), 702–722. 10.1039/D0NP00077A 33404035

[bib30] Grigoriev I. V., Nikitin R., Haridas S., Kuo A., Ohm R., Otillar R., Riley R., Salamov A., Zhao X., Korzeniewski F., Smirnova T., Nordberg H., Dubchak I., Shabalov I. (2014). MycoCosm portal: Gearing up for 1000 fungal genomes. Nucleic Acids Research, 42(D1), D699–D704. 10.1093/nar/gkt1183 24297253 PMC3965089

[bib31] Hanif M., Pardo A. G., Gorfer M., Raudaskoski M. (2002). T-DNA transfer and integration in the ectomycorrhizal fungus *Suillus bovinus* using hygromycin B as a selectable marker. Current Genetics, 41(3), 183–188. 10.1007/s00294-002-0297-8 12174821

[bib32] Hartley A. J., de Mattos-Shipley K., Collins C. M., Kilaru S., Foster G. D., Bailey A. M. (2009). Investigating pleuromutilin-producing *Clitopilus* species and related basidiomycetes. FEMS Microbiology Letters, 297(1), 24–30. 10.1111/j.1574-6968.2009.01656.x 19527297

[bib33] Herzog R., Solovyeva I., Bölker M., Lugones L. G., Hennicke F. (2019). Exploring molecular tools for transformation and gene expression in the cultivated edible mushroom *Agrocybe aegerita*. Molecular Genetics and Genomics, 294(3), 663–677. 10.1007/s00438-018-01528-6 30778675

[bib34] Honda Y., Tanigawa E., Tsukihara T., Nguyen D. X., Kawabe H., Sakatoku N., Watari J., Sato H., Yano S., Tachiki T., Irie T., Watanabe T., Watanabe T. (2019). Stable and transient transformation, and a promoter assay in the selective lignin-degrading fungus, *Ceriporiopsis subvermispora*. AMB Express, 9(1), 92. 10.1186/s13568-019-0818-1 31236750 PMC6591348

[bib35] Hu J., Cutrera J., Li S. (2014). The Impact of Non-electrical Factors on Electrical Gene Transfer. In: Li, S., Cutrera, Electroporation Protocols. Methods in Molecular Biology. Vol. 1121, (pp. 47–54.). R. Heller, J. Teissie (Eds). Humana Press, New York, NY. 10.1007/978-1-4614-9632-8_3 PMC4116183 24510810

[bib36] Iacovelli R., Poon F., Haslinger K. (2024). Identification and reconstitution of the first two enzymatic steps for the biosynthesis of bioactive meroterpenoids from *Hericium erinaceus* (Lion's Mane Mushroom). Molecules (Basel, Switzerland), 29(23), 5576. 10.3390/molecules29235576 39683734 PMC11643632

[bib37] Irie T., Sato T., Saito K., Honda Y., Watanabe T., Kuwahara M., Enei H. (2003). Construction of a homologous selectable marker gene for *Lentinula edodes* transformation. Bioscience, Biotechnology, and Biochemistry, 67(9), 2006–2009. 10.1271/bbb.67.2006 14519992

[bib38] Isaka M., Chinthanom P., Kongthong S., Srichomthong K., Choeyklin R. (2013). Lanostane triterpenes from cultures of the basidiomycete *Ganoderma orbiforme* BCC 22324. Phytochemistry, 87, 133–139. 10.1016/j.phytochem.2012.11.022 23280041

[bib39] Janusz G., Pawlik A., Sulej J., Świderska-Burek U., Jarosz-Wilkołazka A., Paszczyński A. (2017). Lignin degradation: Microorganisms, enzymes involved, genomes analysis and evolution. FEMS Microbiology Reviews, 41(6), 941–962. 10.1093/femsre/fux049 29088355 PMC5812493

[bib40] Jan Vonk P., Escobar N., Wösten H. A. B., Lugones L. G., Ohm R. A. (2019). High-throughput targeted gene deletion in the model mushroom *Schizophyllum commune* using pre-assembled Cas9 ribonucleoproteins. Scientific Reports, 9(1), 1–8. 10.1038/s41598-019-44133-2 31113995 PMC6529522

[bib41] Jermy A. (2011). Soil fungi helped ancient plants to make land. Nature Reviews Microbiology, 9(1), 6–6. 10.1038/nrmicro2494 21204312

[bib42] Jia Y., Li Y., Shang H., Luo Y., Tian Y. (2023). Ganoderic Acid A and its amide derivatives as potential anti-cancer agents by regulating the p53-MDM2 pathway: Synthesis and biological evaluation. Molecules (Basel, Switzerland), 28(5), 2374. 10.3390/molecules28052374 36903622 PMC10004777

[bib43] Kajita S., Sugawara S., Miyazaki Y., Nakamura M., Katayama Y., Shishido K., Iimura Y. (2004). Overproduction of recombinant laccase using a homologous expression system in *Coriolus versicolor*. Applied Microbiology and Biotechnology, 66(2), 194–199. 10.1007/s00253-004-1663-x 15480638

[bib44] Kamiya A., Ueshima H., Nishida S., Honda Y., Kamitsuji H., Sato T., Miyamoto H., Sumita T., Izumitsu K., Irie T. (2023). Development of a gene-targeting system using CRISPR/Cas9 and utilization of pyrG as a novel selectable marker in *Lentinula edodes*. FEMS Microbiology Letters, 370(May), 1–6. 10.1093/femsle/fnad042 37173280

[bib45] Kilaru S., Collins C. M., Hartley A. J., Bailey A. M., Foster G. D. (2009). Establishing molecular tools for genetic manipulation of the pleuromutilin-producing fungus *Clitopilus passeckerianus*. Applied and Environmental Microbiology, 75(22), 7196–7204. 10.1128/AEM.01151-09 19767458 PMC2786515

[bib46] Kim J. K., Park Y. J., Kong W. S., Kang H. W. (2010). Highly efficient electroporation-mediated transformation into edible mushroom *Flammulina velutipes*. Mycobiology, 38(4), 331. 10.4489/myco.2010.38.4.331 23956676 PMC3741529

[bib47] Kim K., Leem Y., Kim K., Kim K., Choi H. T. (2002). Transformation of the medicinal basidiomycete *Trametes versicolor* to hygromycin B resistance by restriction enzyme mediated integration. FEMS Microbiology Letters, 209(2), 273–276. 10.1016/S0378-1097(02)00555-4 12007817

[bib48] Kim S., Ha B. S., Ro H. S. (2015). Current technologies and related issues for mushroom transformation. Mycobiology, 43(1), 1–8. 10.5941/MYCO.2015.43.1.1 25892908 PMC4397374

[bib49] Krzyczkowski W., Malinowska E., Herold F. (2010). Erinacine A biosynthesis in submerged cultivation of *Hericium erinaceum* : Quantification and improved cultivation. Engineering in Life Sciences, 10(5), 446–457. 10.1002/elsc.201000084

[bib50] Leavell M. D., McPhee D. J., Paddon C. J. (2016). Developing fermentative terpenoid production for commercial usage. Current Opinion in Biotechnology, 37, 114–119. 10.1016/j.copbio.2015.10.007 26723008

[bib51] Leem Y., Kim S., Ross I. K., Choi H. T. (1999). Transformation and laccase mutant isolation in *Coprinus congregatus* by restriction enzyme-mediated integration. FEMS Microbiology Letters, 172(1), 35–40. 10.1111/j.1574-6968.1999.tb13446.x

[bib52] Li D., Tang Y., Lin J., Cai W. (2017). Methods for genetic transformation of filamentous fungi. Microbial Cell Factories, 16(1), 1–13. 10.1186/s12934-017-0785-7 28974205 PMC5627406

[bib53] Li W., Zhou W., Kim E. J., Shim S. H., Kang H. K., Kim Y. H. (2015). Isolation and identification of aromatic compounds in Lion's Mane Mushroom and their anticancer activities. Food Chemistry, 170, 336–342. 10.1016/j.foodchem.2014.08.078 25306354

[bib54] Liu C., Minami A., Ozaki T., Wu J., Kawagishi H., Maruyama J., Oikawa H. (2019). Efficient reconstitution of basidiomycota diterpene erinacine gene cluster in ascomycota host *Aspergillus oryzae* based on genomic DNA sequences. Journal of the American Chemical Society, 141(39), 15519–15523. 10.1021/jacs.9b08935 31535864

[bib55] Liu J., Cui H., Wang R., Xu Z., Yu H., Song C., Lu H., Li Q., Xing D., Tan Q., Sun W., Zou G., Shang X. (2022). A simple and efficient CRISPR/Cas9 system using a ribonucleoprotein method for *Flammulina filiformis*. Journal of Fungi, 8(10), 1000. 10.3390/jof8101000 36294565 PMC9604558

[bib56] Lorito M., Hayes C. K., Di Pietro A., Harman G. E. (1993). Biolistic transformation of *Trichoderma harzianum* and *Gliocladium virens* using plasmid and genomic DNA. Current Genetics, 24(4), 349–356. 10.1007/BF00336788 8252645

[bib57] Lugones L. G., Scholtmeijer K., Klootwijk R., Wessels J. G. H. (1999). Introns are necessary for mRNA accumulation in *Schizophyllum commune*. Molecular Microbiology, 32(4), 681–689. 10.1046/j.1365-2958.1999.01373.x 10361273

[bib58] Luo P., Huang J.-H., Lv J.-M., Wang G.-Q., Hu D., Gao H. (2024). Biosynthesis of fungal terpenoids. Natural Product Reports, 41(5), 748–783. 10.1039/D3NP00052D 38265076

[bib59] Ma B.-J., Yu H.-Y., Shen J.-W., Ruan Y., Zhao X., Zhou H., Wu T.-T. (2010). Cytotoxic aromatic compounds from *Hericium erinaceum*. The Journal of Antibiotics, 63(12), 713–715. 10.1038/ja.2010.112 20924382

[bib60] Ma L., Chen H., Dong P., Lu X. (2013). Anti-inflammatory and anticancer activities of extracts and compounds from the mushroom *Inonotus obliquus*. Food Chemistry, 139(1-4), 503–508. 10.1016/j.foodchem.2013.01.030 23561137

[bib61] Magaña-Ortíz D., Coconi-Linares N., Ortiz-Vazquez E., Fernández F., Loske A. M., Gómez-Lim M. A. (2013). A novel and highly efficient method for genetic transformation of fungi employing shock waves. Fungal Genetics and Biology, 56, 9–16. 10.1016/j.fgb.2013.03.008 23583899

[bib62] Maróstica M. R., Pastore G. M. (2007). Tropical fruit flavour. In Flavours and Fragrances(pp. 189–201.). Springer, Berlin Heidelberg. 10.1007/978-3-540-49339-6_8

[bib63] Maruyama J. I., Kitamoto K. (2008). Multiple gene disruptions by marker recycling with highly efficient gene-targeting background (ΔligD) in *Aspergillus oryzae*. Biotechnology Letters, 30(10), 1811–1817. 10.1007/s10529-008-9763-9 18574559

[bib64] Maza P. A. M. A., Lee J.-H., Kim Y.-S., Sun G.-M., Sung Y.-J., Ponomarenko L. P., Stonik V. A., Ryu M., Kwak J.-Y. (2021). Inotodiol from *Inonotus obliquus* chaga mushroom induces atypical maturation in dendritic cells. Frontiers in Immunology, 12, 650841. 10.3389/fimmu.2021.650841 33777049 PMC7994266

[bib65] Meyer V. (2021). Metabolic engineering of filamentous fungi. In Metabolic Engineering(Vol. 13, pp. 765–801.). Wiley. 10.1002/9783527823468.ch20

[bib66] Meyer V., Basenko E. Y., Benz J. P., Braus G. H., Caddick M. X., Csukai M., de Vries R. P., Endy D., Frisvad J. C., Gunde-Cimerman N., Haarmann T., Hadar Y., Hansen K., Johnson R. I., Keller N. P., Kraševec N., Mortensen U. H., Perez R., Ram A. F. J., Wösten H. A. B. (2020). Growing a circular economy with fungal biotechnology: A white paper. Fungal Biology and Biotechnology, 7(1), 5. 10.1186/s40694-020-00095-z 32280481 PMC7140391

[bib67] Michielse C. B., Hooykaas P. J. J., van den Hondel C. A. M. J. J., Ram A. F. J. (2008). *Agrobacterium*-mediated transformation of the filamentous fungus *Aspergillus awamori*. Nature Protocols, 3(10), 1671–1678. 10.1038/nprot.2008.154 18833205

[bib68] Money N. P. (2016). Fungi and biotechnology. S.C. Watkinson, L. Boddy, N. Money (Eds.). The Fungi: Third Edition(pp. 401–424.). Elsevier. 10.1016/B978-0-12-382034-1.00012-8

[bib69] Moss G. P., Smith P. A. S., Tavernier D. (1995). Glossary of class names of organic compounds and reactivity intermediates based on structure (IUPAC Recommendations 1995). Pure and Applied Chemistry, 67(8-9), 1307–1375. 10.1351/pac199567081307

[bib70] Mosunova O., Navarro-Muñoz J. C., Collemare J. (2020). The biosynthesis of fungal secondary metabolites: From fundamentals to biotechnological applications. In Reference Module in Life Sciences(pp. 1–19.). Elsevier. 10.1016/B978-0-12-809633-8.21072-8

[bib71] Nakazawa T., Inoue C., Nguyen D. X., Kawauchi M., Sakamoto M., Honda Y. (2022). CRISPR/Cas9 using a transient transformation system in *Ceriporiopsis subvermispora*. Applied Microbiology and Biotechnology, 106(17), 5575–5585. 10.1007/s00253-022-12095-7 35902408

[bib73] Nielsen J. C., Nielsen J. (2017). Development of fungal cell factories for the production of secondary metabolites: Linking genomics and metabolism. Synthetic and Systems Biotechnology, 2(1), 5–12. 10.1016/j.synbio.2017.02.002 29062956 PMC5625732

[bib74] Niu X.-M., Li S.-H., Sun H.-D., Che C.-T. (2006). Prenylated phenolics from *Ganoderma fornicatum*. Journal of Natural Products, 69(9), 1364–1365. 10.1021/np060218k 16989537

[bib75] Ohya N., Takizawa J., Kawahara S., Tanaka Y. (1998). Molecular weight distribution of polyisoprene from *Lactarius volemus*. Phytochemistry, 48(5), 781–786. 10.1016/S0031-9422(97)00829-7

[bib76] Pan X., Rudolf J. D., Dong L.-B. (2024). Class II terpene cyclases: Structures, mechanisms, and engineering. Natural Product Reports, 41(3), 402–433. 10.1039/D3NP00033H 38105714 PMC10954422

[bib77] Paterson R. R. M. (2006). *Ganoderma*—A therapeutic fungal biofactory. Phytochemistry, 67(18), 1985–2001. 10.1016/j.phytochem.2006.07.004 16905165

[bib78] Pohl C., Kiel J. A. K. W., Driessen A. J. M., Bovenberg R. A. L., Nygård Y. (2016). CRISPR/Cas9 based genome editing of *Penicillium chrysogenum*. ACS Synthetic Biology, 5(7), 754–764. 10.1021/acssynbio.6b00082 27072635

[bib79] Punt P. J., van Biezen N., Conesa A., Albers A., Mangnus J., van den Hondel C. (2002). Filamentous fungi as cell factories for heterologous protein production. Trends in Biotechnology, 20(5), 200–206. 10.1016/S0167-7799(02)01933-9 11943375

[bib80] Qin H., Xiao H., Zou G., Zhou Z., Zhong J.-J. (2017). CRISPR-Cas9 assisted gene disruption in the higher fungus *Ganoderma* species. Process Biochemistry, 56, 57–61. 10.1016/j.procbio.2017.02.012

[bib81] Rapior S., Cavalié S., Croze P., Andary C., Pélissier Y., Bessière J.-M. (1996). Volatile components of ten frozen mushrooms (Basidiomycetes). Journal of Essential Oil Research, 8(1), 63–66. 10.1080/10412905.1996.9700556

[bib82] Rizzo G., Goggi S., Giampieri F., Baroni L. (2021). A review of mushrooms in human nutrition and health. Trends in Food Science & Technology, 117, 60–73. 10.1016/j.tifs.2020.12.025

[bib83] Salame T. M., Knop D., Tal D., Levinson D., Yarden O., Hadar Y. (2012). Predominance of a versatile-peroxidase-encoding gene, mnp4, as demonstrated by gene replacement via a gene targeting system for *Pleurotus ostreatus*. Applied and Environmental Microbiology, 78(15), 5341–5352. 10.1128/AEM.01234-12 22636004 PMC3416422

[bib84] Salehi B., Upadhyay S., Erdogan Orhan I., Kumar Jugran A., L.D. Jayaweera S., A. Dias D., Sharopov F., Taheri Y., Martins N., Baghalpour N., C. Cho W., Sharifi-Rad J. (2019). Therapeutic potential of α- and β-pinene: A miracle gift of nature. Biomolecules, 9(11), 738. 10.3390/biom9110738 31739596 PMC6920849

[bib85] Santiago Y., Chan E., Liu P. Q., Orlando S., Zhang L., Urnov F. D., Holmes M. C., Guschin D., Waite A., Miller J. C., Rebar E. J., Gregory P. D., Klug A., Collingwood T. N. (2008). Targeted gene knockout in mammalian cells by using engineered zinc-finger nucleases. Proceedings of the National Academy of Sciences, 105(15), 5809–5814. 10.1073/pnas.0800940105 PMC2299223 18359850

[bib86] Schmidt-Dannert C. (2015). Biosynthesis of terpenoid natural products in fungi. Advances in Biochemical Engineering/Biotechnology, 148, 19–61. 10.1007/10_2014_283 25414054

[bib87] Schobert R., Knauer S., Seibt S., Biersack B. (2011). Anticancer active illudins: Recent developments of a potent alkylating compound class. Current Medicinal Chemistry, 18(6), 790–807. 10.2174/092986711794927766 21182482

[bib88] Sharma R. (2017). Ectomycorrhizal mushrooms: Their diversity, ecology and practical applications. In Mycorrhiza—Function, Diversity, State of the Art(pp. 99–131.). Springer International Publishing. 10.1007/978-3-319-53064-2_7

[bib89] Shi L., Chen D., Xu C., Ren A., Yu H., Zhao M. (2017). Highly-efficient liposome-mediated transformation system for the basidiomycetous fungus *Flammulina velutipes*. The Journal of General and Applied Microbiology, 63(3), 179–185. 10.2323/jgam.2016.10.003 28484117

[bib90] Steindorff A. S., Aguilar-Pontes M. V., Robinson A. J., Andreopoulos B., LaButti K., Kuo A., Mondo S., Riley R., Otillar R., Haridas S., Lipzen A., Grimwood J., Schmutz J., Clum A., Reid I. D., Moisan M.-C., Butler G., Nguyen T. T. M., Dewar K., Grigoriev I. V. (2024). Comparative genomic analysis of thermophilic fungi reveals convergent evolutionary adaptations and gene losses. Communications Biology, 7(1), 1124. 10.1038/s42003-024-06681-w 39266695 PMC11393059

[bib91] Sugano S. S., Suzuki H., Shimokita E., Chiba H., Noji S., Osakabe Y., Osakabe K. (2017). Genome editing in the mushroom-forming basidiomycete *Coprinopsis cinerea*, optimized by a high-throughput transformation system. Scientific Reports, 7(1), 1–9. 10.1038/s41598-017-00883-5 28455526 PMC5430836

[bib92] Sun L., Cai H., Xu W., Hu Y., Gao Y., Lin Z. (2001). Efficient transformation of the medicinal mushroom *Ganoderma lucidum*. Plant Molecular Biology Reporter, 19(4), 383–384. 10.1007/bf02772841

[bib93] Sun S., Ai L., Zhang H., Weng C., Lai C., Liu L. (2017). Enhanced production of pleuromutilin by *Pleurotus mutilus* and study on its molecular structure. Food Chemistry, 230, 350–353. 10.1016/j.foodchem.2017.03.064 28407921

[bib94] Tanaka Y., Kawahara S., Eng A. H., Takei A., Ohya N. (1994). Structure of cis-polyisoprene from *Lactarius* mushrooms. Acta Biochimica Polonica, 41(3), 303–309. 10.18388/abp.1994_4719 7856401

[bib95] Tang Y.-J., Zhong J.-J. (2002). Fed-batch fermentation of *Ganoderma lucidum* for hyperproduction of polysaccharide and ganoderic acid. Enzyme and Microbial Technology, 31(1-2), 20–28. 10.1016/S0141-0229(02)00066-2

[bib96] Tao Q., Ma K., Yang Y., Wang K., Chen B., Huang Y., Han J., Bao L., Liu X.-B., Yang Z., Yin W.-B., Liu H. (2016). Bioactive sesquiterpenes from the edible mushroom *Flammulina velutipes* and their biosynthetic pathway confirmed by genome analysis and chemical evidence. The Journal of Organic Chemistry, 81(20), 9867–9877. 10.1021/acs.joc.6b01971 27684789

[bib97] Tu J. L., Bai X. Y., Xu Y. L., Li N., Xu J. W. (2021). Targeted gene insertion and replacement in the basidiomycete *Ganoderma lucidum* by inactivation of nonhomologous end joining using CRISPR/Cas9. Applied and Environmental Microbiology, 87(23), 1–11. 10.1128/AEM.01510-21 PMC8579997 34524900

[bib98] van de Rhee M. D., Graça P. M. A., Huizing H. J., Mooibroek H. (1996). Transformation of the cultivated mushroom, *Agaricus bisporus*, to Hygromycin B resistance. MGG Molecular & General Genetics, 250(3), 252. 10.1007/s004380050074 8602139

[bib99] Van Peer A. F., De Bekker C., Vinck A., Wösten H. A. B., Lugones L. G. (2009). Phleomycin increases transformation efficiency and promotes single integrations in *Schizophyllum commune*. Applied and Environmental Microbiology, 75(5), 1243–1247. 10.1128/AEM.02162-08 19114524 PMC2648172

[bib100] van Santen J. A., Poynton E. F., Iskakova D., McMann E., Alsup T. A., Clark T. N., Fergusson C. H., Fewer D. P., Hughes A. H., McCadden C. A., Parra J., Soldatou S., Rudolf J. D., Janssen E. M.-L., Duncan K. R., Linington R. G. (2022). The Natural Products Atlas 2.0: A database of microbially-derived natural products. Nucleic Acids Research, 50(D1), D1317–D1323. 10.1093/nar/gkab941 34718710 PMC8728154

[bib101] Wang L., Li J., Zhang J., Li Z., Liu H., Wang Y. (2020). Traditional uses, chemical components and pharmacological activities of the genus *Ganoderma* P. Karst.: A review. RSC Advances, 10(69), 42084–42097. 10.1039/D0RA07219B 35516772 PMC9057998

[bib102] Wang Q., Cao R., Zhang Y., Qi P., Wang L., Fang S. (2021). Biosynthesis and regulation of terpenoids from basidiomycetes: Exploration of new research. AMB Express, 11(1), 150. 10.1186/s13568-021-01304-7 34779947 PMC8594250

[bib103] Wang Q., Coleman J. J. (2019). Progress and challenges: Development and implementation of CRISPR/Cas9 technology in filamentous fungi. Computational and Structural Biotechnology Journal, 17, 761–769. 10.1016/j.csbj.2019.06.007 31312414 PMC6607083

[bib104] Wang S., Han J., Xia J., Hu Y., Shi L., Ren A., Zhu J., Zhao M. (2020). Overexpression of nicotinamide mononucleotide adenylyltransferase (nmnat) increases the growth rate, Ca2+ concentration and cellulase production in *Ganoderma lucidum*. Applied Microbiology and Biotechnology, 104(16), 7079–7091. 10.1007/s00253-020-10763-0 32632475

[bib105] Wang T., Yue S., Jin Y., Wei H., Lu L. (2021). Advances allowing feasible pyrG gene editing by a CRISPR-Cas9 system for the edible mushroom *Pleurotus eryngii*. Fungal Genetics and Biology, 147, 103509. 10.1016/j.fgb.2020.103509 33400990

[bib106] Wawrzyn G. T., Quin M. B., Choudhary S., López-Gallego F., Schmidt-Dannert C. (2012). Draft genome of *Omphalotus olearius* provides a predictive framework for sesquiterpenoid natural product biosynthesis in Basidiomycota. Chemistry & Biology, 19(6), 772–783. 10.1016/j.chembiol.2012.05.012 22726691 PMC3383649

[bib107] Wen B., Huang H., Lu L., Liu T., Liu R. (2025). Overexpression of geranylgeranyl diphosphate synthase and cyclase enhances pleuromutilin production in *Clitopilus passeckerianus* T6. Biotechnology Journal, 20(2), 1–9. 10.1002/biot.202500004 40012214

[bib108] Xiao H., Zhong J. J. (2016). Production of useful terpenoids by higher-fungus cell factory and synthetic biology approaches. Trends in Biotechnology, 34(3), 242–255. 10.1016/j.tibtech.2015.12.007 26787340

[bib109] Yang L., Lübeck M., Lübeck P. S. (2017). *Aspergillus* as a versatile cell factory for organic acid production. Fungal Biology Reviews, 31(1), 33–49. 10.1016/j.fbr.2016.11.001

[bib110] Yang Y., Zhang S., Ma K., Xu Y., Tao Q., Chen Y., Chen J., Guo S., Ren J., Wang W., Tao Y., Yin W., Liu H. (2017). Discovery and characterization of a new family of diterpene cyclases in bacteria and fungi. Angewandte Chemie International Edition, 56(17), 4749–4752. 10.1002/anie.201700565 28371074

[bib111] Yao D., Ma Y., Ran J., Wang J., Kües U., Liu J., Zhou D., Zhang X., Fang Z., Xiao Y. (2024). Enhanced extracellular production of laccase in *Coprinopsis cinerea* by silencing chitinase gene. Applied Microbiology and Biotechnology, 108(1), 324. 10.1007/s00253-024-13164-9 38713211 PMC11076350

[bib112] Yılmaz T. M., Mungan M. D., Berasategui A., Ziemert N. (2023). FunARTS, the fungal bioActive compound Resistant Target Seeker, an exploration engine for target-directed genome mining in fungi. Nucleic Acids Research, 51(W1), W191–W197. 10.1093/nar/gkad386 37207330 PMC10320164

[bib113] Yin X., Feng T., Li Z.-H., Dong Z.-J., Li Y., Liu J. K. (2013). Highly oxygenated meroterpenoids from fruiting bodies of the mushroom *Tricholoma terreum*. Journal of Natural Products, 76(7), 1365–1368. 10.1021/np400359y 23837944

[bib114] Yoo N.-H., Kim J.-P., Yun B.-S., Ryoo I.-J., Lee I.-K., Yoon E.-S., Koshino H., Yoo I.-D. (2006). Hirsutenols D, E and F, New Sesquiterpenes from the culture broth of *Stereum hirsutum*. The Journal of Antibiotics, 59(2), 110–113. 10.1038/ja.2006.16 16629412

[bib115] Yuan W., Jiang C., Wang Q., Fang Y., Wang J., Wang M., Xiao H. (2022). Biosynthesis of mushroom-derived type II ganoderic acids by engineered yeast. Nature Communications, 13(1), 7740. 10.1038/s41467-022-35500-1 PMC9748899 36517496

[bib116] Zhang D. H., Jiang L. X., Li N., Yu X., Zhao P., Li T., Xu J. W. (2017). Overexpression of the squalene epoxidase gene alone and in combination with the 3-hydroxy-3-methylglutaryl coenzyme A gene increases ganoderic acid production in *Ganoderma lingzhi*. Journal of Agricultural and Food Chemistry, 65(23), 4683–4690. 10.1021/acs.jafc.7b00629 28530827

[bib117] Zhang J. J., Shi L., Chen H., Sun Y. Q., Zhao M. W., Ren A., Chen M. J., Wang H., Feng Z. Y. (2014). An efficient *Agrobacterium*-mediated transformation method for the edible mushroom *Hypsizygus marmoreus*. Microbiological Research, 169(9-10), 741–748. 10.1016/j.micres.2014.01.004 24612605

[bib118] Zhang S., Shi G., Xu X., Guo X., Li S., Li Z., Wu Q., Yin W. B. (2024). Global analysis of natural products biosynthetic diversity encoded in fungal genomes. Journal of Fungi, 10(9), 653. 10.3390/jof10090653 39330413 PMC11433233

[bib119] Zhao F. Y., Lin J. F., Zeng X. L., Guo L. Q., Wang Y. H., You L. R. (2010). Improvement in fruiting body yield by introduction of the *Ampullaria crossean* multi-functional cellulase gene into Volvariella volvacea. Bioresource Technology, 101(16), 6482–6486. 10.1016/j.biortech.2010.03.035 20378340

